# Clinical Presentation, Detection, and Immunopathogenesis of *Mycoplasma hyosynoviae* Field Isolates in Experimentally Inoculated Pigs

**DOI:** 10.3390/pathogens15010066

**Published:** 2026-01-08

**Authors:** Nubia R. Macedo, Bailey L. Arruda, Luis G. Giménez-Lirola, Ganwu Li, Locke Karriker, Jordi Mora, María J. Clavijo

**Affiliations:** 1 Veterinary Diagnostic and Production Animal Medicine, College of Veterinary Medicine, Iowa State University, Ames, IA 50011, USA; nubia@iastate.edu (N.R.M.); bailey.arruda@usda.gov (B.L.A.);; 2 Swine Medicine Education Center, College of Veterinary Medicine, Iowa State University, Ames, IA 50011, USA; 3 Eco Animal Health, The Grange 100 High Street, Southgate, London N14 6BN, UK

**Keywords:** *Mycoplasma hyosynoviae*, swine lameness, swine arthritis, virulence, pathogenesis, host immune response, experimental infection

## Abstract

*Mycoplasma hyosynoviae* is a significant pathogen in swine populations, contributing to polyarthritis and lameness in growing pigs. This study characterizes the clinical presentation, pathogen detection, immune response, and lesion development following experimental inoculation with two distinct *M. hyosynoviae* strains. Pigs were inoculated with either a low- or high-virulence strain and monitored for 18 days. Lameness was observed throughout the study, with affected pigs exhibiting mild to moderate clinical signs. *M. hyosynoviae* was often detected in the tonsils, while detection in oral fluids was transient. Serum IgG levels increased significantly in the inoculated groups. IL-1β, IL-6, and TNF-α cytokines were elevated only at 7 DPI, whereas IL-8, IL-10, and IFN-γ levels were unchanged in both inoculated groups. Notably, only pigs inoculated with the high-virulence strain developed lesions, and *M. hyosynoviae* was detected only in the synovial fluid by PCR from this group. These findings highlight strain-dependent differences in the pathogenesis of *M. hyosynoviae*. The pathological differences between these strains suggest variations in adherence factors, immune evasion capabilities, or metabolic adaptability. Further research is warranted to elucidate the genetic determinants of virulence and the protective role of humoral and cellular immune responses in *M. hyosynoviae* infection.

## 1. Introduction

*Mycoplasma hyosynoviae* (*M. hyosynoviae*) is a significant pathogen in swine production which is responsible for infectious polyarthritis in growing pigs, often leading to substantial economic losses [1,2]. *M. hyosynoviae* colonizes the upper respiratory tract, particularly the tonsils, where it can persist asymptomatically and establish long-term carriage [3,4,5]. *M. hyosynoviae* spreads systemically, establishing joint infection and causing clinical disease characterized by lameness, joint swelling, and pain [4,6]. Disease onset typically occurs in pigs aged 3 to 6 months, coinciding with the loss of maternal immunity and exposure to various management-related stressors [7,8].

The progression of *M. hyosynoviae*-associated arthritis is influenced by known risk factors, including immune suppression, physical stressors (e.g., regrouping, mixing, transport), genetic susceptibility, and high-virulence strains [8,9,10,11]. Poor acclimation practices and suboptimal biosecurity may also facilitate earlier or more severe disease onset due to host immunity, increased variability among field strains, and environmental conditions [7,8,9,10,11,12,13,14,15]. Despite its recognition as a significant pathogen, critical gaps remain in understanding its pathogenesis, host–pathogen interactions, and mechanisms of persistent colonization. These challenges are compounded by the lack of reliable commercial vaccines, leaving antimicrobial therapy and autogenous vaccines as the primary control measures [1]. However, effective treatment depends heavily on early detection and intervention. Delayed diagnosis often results in chronic lameness, reduced productivity, and increased culling rates.

The actual burden of *M. hyosynoviae*-associated arthritis remains difficult to quantify, owing to diagnostic complexity, inconsistent case submissions, and overlap with other non-infectious and infectious causes of lameness such as *Mycoplasma hyorhinis*, *Glaesserella parasuis*, and *Erysipelothrix rhusiopathiae* [2,14]. Although field reports consistently identify *M. hyosynoviae* as a frequent contributor to lameness in grow-finish pigs and a potential factor in sow mortality [15,16], a recent diagnostic surveillance analysis revealed a contrasting trend. Specifically, despite an overall rise in arthritis submissions during the study period, *M. hyosynoviae*-associated diagnoses declined over time, coinciding with a reduction in PCR testing for this agent [17]. This may reflect decreased sample submissions from older pigs, where the pathogen is most commonly detected, or successful mitigation through antimicrobial usage [1,10]. Alternatively, the decline may stem from diagnostic limitations, especially in cases of chronic or polymicrobial infections, where the pathogen is more difficult to confirm [2].

A high variability among *M. hyosynoviae* field strains has been reported [9], but very little is known about the pathogenesis of different *M. hyosynoviae* strains. Therefore, our goal is to investigate the different clinical manifestations, pathogen detection patterns, lesion progression, immune responses, and joint pathology in pigs experimentally inoculated with two genetically distinct strains of *M. hyosynoviae*, hypothesizing that significant variances in the results will be observed.

## 2. Materials and Methods

### 2.1. Experimental Design

Sixteen eight-week-old cesarean-derived, colostrum-deprived (CDCD) pigs were obtained from Struve Labs International (Manning, IA, USA). Pigs were housed at the Iowa State University animal research facility and acclimated for five days before inoculation. Upon arrival, all pigs’ tonsils tested negative by PCR for *M. hyosynoviae* and *M. hyorhinis*, and serum negative for porcine reproductive and respiratory syndrome virus (PRRSV) by PCR.

Upon arrival, pigs were classified by litter, weight, and sex. Then, the pigs were assigned to different rooms representing three groups: (1) negative control (*n* = 4), (2) high-virulence *M. hyosynoviae* (*n* = 6), and (3) low-virulence *M. hyosynoviae* (*n* = 6). Within each room, pigs were individually penned. The negative control group (*n* = 4) was sham-inoculated with a culture medium. In contrast, the other two groups were inoculated with either 10^8^ CFU/mL of a low-virulence or high-virulence *M. hyosynoviae* strain, as previously described [12] (Table 1). Inoculation was performed via intranasal (1 mL per nostril), tonsillar painting (2 mL), and intravenous (1 mL) routes. Pigs were monitored daily for clinical signs, humanely euthanized at 16 days post-inoculation (DPI), and evaluated for macroscopic and microscopic lesions. All procedures were approved by the Iowa State University Office for Responsible Research and Institutional Animal Care and Use Committee.

### 2.2. Animal Housing

Pigs were housed in a BSL-2 isolation facility specifically designed for research on livestock infectious diseases. The facility was equipped with a non-recirculating ventilation system to prevent the transmission of aerosols between rooms. Personnel followed strict personal protective equipment (PPE) and room-specific entry protocols to prevent cross-contamination. All rooms and equipment were disinfected before animal entry and maintained under BSL-2 containment procedures throughout the study.

### 2.3. Mycoplasma hyosynoviae Strains and Inoculation Routes

**Figure 1:**
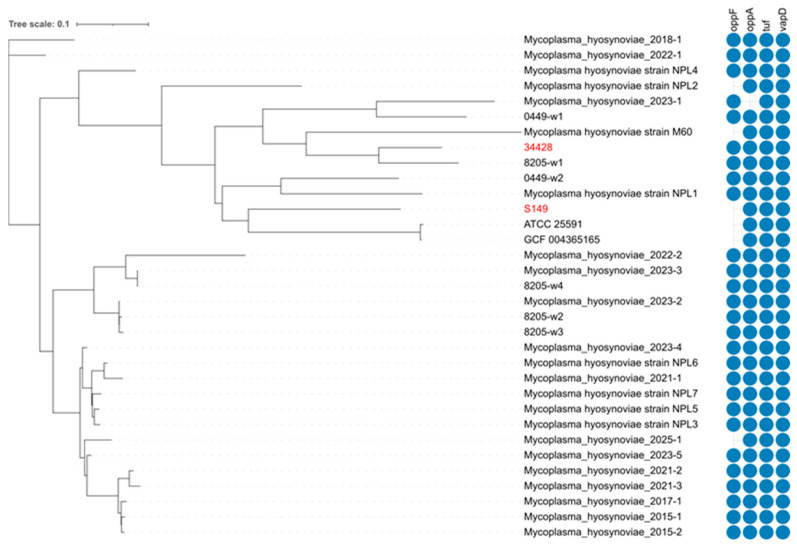
SNP-based phylogenetic and genomic comparison of *Mycoplasma hyosynoviae* isolates. Whole-genome SNP analysis showing the genetic relationship between the two isolates from this study (highlighted in red) and previously detected clinical cases from different sample matrices submitted to the ISU Veterinary Diagnostic Laboratory (ISU-VDL). Blue circles indicate shared virulence-associated genes. Reference genome sequences were retrieved from NCBI: NPL1 (JFKL01000049.1), NPL2 (JFKK01000042.1), NPL3 (JFKJ01000029.1), NPL4 (JFKI01000009.1), NPL5 (JFKM01000003.1), NPL6 (JFKH01000034.1), NPL7 (JFKG01000007.1), M60 (CP008748), and *M. hyosynoviae* Ross and Karmon [S16] 25591^TM^ (NZ_SOCH01000000) (Supplementary Table S1).

Two distinct *M. hyosynoviae* strains were used in this study. The first strain (S149) was obtained from swine joint fluid and had been previously characterized as low virulent [11,12,18]. The second strain (34428) was a field isolate collected in 2014 from a 15-week-old pig with arthritis, as determined by joint fluid culture at the Iowa State University Veterinary Diagnostic Laboratory (ISU VDL). This strain was classified as highly virulent based on reports from the originating farm, which experienced lameness affecting over 45% of the finishing population (Figure 1). Additionally, the submitted pig exhibited severe arthritis, further supporting the virulence classification [15].

The preparation of *M. hyosynoviae* inoculum followed a previously described protocol [12]. Briefly, frozen stocks of each of the *M. hyosynoviae* strains were thawed at room temperature (RT; 20–24 °C), and 0.5 mL was used to inoculate 5 mL of Pleuropneumonia-like organism (PPLO) medium (Difco; BD Biosciences, Franklin Lakes, NJ, USA) supplemented with 15% horse serum (Atlanta Biologicals, Flowery Branch, GA, USA). After 24 h of incubation at 37 °C, the culture was expanded by transferring 5 mL into 25 mL of fresh PPLO medium and incubating for an additional 24 h at 37 °C.

Quantification was performed by serial dilution in PPLO broth. The inoculum was tested by PCR to confirm the purity of *M. hyosynoviae* and exclude contamination with *M. hyorhinis*, *M. hyopneumoniae*, and *M. flocculare*. Additionally, an aliquot was plated on blood agar and incubated at 37 °C for 72 h to rule out bacterial contamination. 

Intranasal administration was performed using a 1 mL aliquot of inoculum, delivered into each nostril (totaling 2 mL per pig), via a sterile syringe without a needle. This method aimed to mimic respiratory exposure and establish upper airway colonization. Tonsillar painting involved the application of 2 mL of inoculum directly onto the tonsillar area using a sterile cotton-tipped swab, ensuring local mucosal contact and enhancing the likelihood of oropharyngeal colonization. For intravenous inoculation, a butterfly catheter (23 G or 25 G) was inserted into the lateral ear vein under manual restraint. A 1 mL dose of inoculum was slowly injected to promote systemic dissemination of the pathogen. All inoculations were performed on Day 0, and animals were monitored immediately post-inoculation for adverse reactions. This multimodal approach was designed to reflect mucosal and systemic phases of *M. hyosynoviae* infection and ensure reproducibility across animals [18,19].

### 2.4. Sequencing, Phylogenetic Analysis, and Virulence Gene Identification

Total nucleic acids were extracted using the ChargeSwitch gDNA mini bacteria kit (Life Technologies). Libraries were prepared using the Nextera XT kit (Illumina) and sequenced on an Illumina MiSeq with the 500-cycle v2 kit to generate 250 bp paired-end reads. Demultiplexing was performed automatically on the MiSeq using default settings.

Bioinformatics analysis was performed as described previously [20,21,22]. Briefly, raw reads were trimmed to remove adapters and low-quality bases, then assembled using SPAdes with paired-end and mismatch correction options. Virulence genes were identified using SRST2 with the VFDB database [23]. Assembled contigs were also BLASTed against VFDB, and hits with an identity of greater than 70% and coverage of greater than 50% were combined with SRST2 results. The phylogenetic tree was constructed using kSNP3 with a k-mer size of 19. To assess the reliability of the inferred relationships, we generated an additional genetic distance matrix using VCF2D, which is based on the SNP sites identified by kSNP3. This independent distance matrix was then used to cross-validate the genetic distances and overall topology of the phylogenetic tree. Phylogenetic trees and molecular features were visualized using iTOL v7.

### 2.5. Clinical Evaluation

Pigs were monitored every other DPI for clinical signs, including lameness, joint swelling, and changes in rectal temperature. Lameness assessments were conducted on a flat, firm surface while pigs were observed walking and standing. Each pig was assigned a lameness score based on a previously established score system [24] (Table 2).

### 2.6. Macroscopic and Microscopic Evaluation

At the conclusion of the study, all pigs were euthanized using a penetrating captive bolt (Accles and Shelvoke, Ltd., Sutton Coalfield, UK), followed by exsanguination. 

The stifle, hock, and elbow joints of both the right and left sides were assessed for macroscopic and microscopic lesions by a diagnostic pathologist who was blinded to the treatment group. Synovial sections from each joint were placed in formalin and processed routinely for microscopic evaluation. A macroscopic and microscopic arthritis/synovitis severity score was assigned to each examined joint. Each joint was individually evaluated for synovial hyperplasia/hyperemia, fibrin deposition, and increased synovial fluid. Lesions were assigned a score from 0 to 3, where 0 indicated no visible lesions, 1 represented mild synovial hyperplasia/hyperemia and/or mild increase in synovial fluid without fibrin, 2 showed moderate synovial hyperplasia/hyperemia, and/or synovial fluid accumulation with or without fibrin, and 3 denoted severe synovial hyperplasia/hyperemia with fibrinous exudate. The total gross lesion score per pig was calculated by summing the scores from all assessed joints. For microscopic analysis, the average number of leukocytes observed in the three most severely affected high-power fields (400× magnification) per tissue section was calculated. A composite leukocyte count for each pig was generated by summing the lesion scores across all sampled joint sites. Moreover, the average of the hock, stifle, and elbow joint score values from the different groups was also evaluated.

### 2.7. Sample Collection, Processing, and Testing

Tonsil swabs, individual oral fluid samples, and serum samples were collected at the time points outlined in Table 3. At necropsy, synovial fluid from each joint was aseptically collected using a needle and syringe. Tonsil swabs were collected using rayon swabs with Amies broth (BD BBL^TM^ CultureSwab^TM^ Transport System with liquid Amies, Thermo Fisher Scientific, Pittsburgh, PA, USA). Oral fluids (one rope/pig) were obtained by suspending cotton ropes (Web Rigging Supply, Inc., Lake Barrington, IL, USA) in each pen for 30 min, allowing pigs to chew them. Fluids were extracted from the ropes by mechanical compression and transferred to 50 mL centrifuge tubes (Corning^®^ Falcon^®^ Centrifuge Tubes, MiliporeSigma, St. Louis, MO, USA) [25]. All samples were stored at −80 °C until tested for *M. hyosynoviae* qPCR to confirm the presence of infection.

Nucleic acid extraction from tonsil, oral fluid, and synovial fluid samples was performed using the MagMAXTM Pathogen RNA/DNA kit (Applied Biosystems, Life Technologies, Carlsbad, CA, USA) and the Thermo Scientific Kingfisher Flex automated magnetic particle processor (Thermo Fisher Scientific, Pittsburgh, PA, USA), following the manufacturer’s instructions. The *M. hyosynoviae* qPCR targeted the 16S ribosomal DNA, and was performed as previously described [15,26]. Briefly, each reaction contained 0.1 mM of forward and reverse primers, 12.5 µL of Qiagen Quantitect SYBR Green (Qiagen, Hilden, Germany), and 2.5 µL of extracted DNA. PCR amplification was conducted on an AB 7500 Fast instrument (Applied Biosystem^®^, Foster City, CA, USA) under the following cycling conditions: 1 cycle of 95 °C for 15 min, 45 cycles of 94 °C for 15 s, and 60 °C for 1 min. Samples with quantification cycle (Cq) value < 44 were considered positive for *M. hyosynoviae*.

Blood samples were collected from the jugular vein or cranial vena cava using blood collection tubes (BD Vacutainer blood collection serum tubes, Franklin Lakes, NJ, USA). Samples were centrifuged at 1500× *g* for 15 min, and serum was stored at −20 °C until further analysis for antibodies and cytokines.

### 2.8. M. hyosynoviae Surface Protein-Based ELISA

An indirect ELISA was used to detect IgG antibodies in serum [15]. Briefly, 96-well plates were coated with 2–3 µg/well of *M. hyosynoviae* strain 34428 surface proteins extracted using a Tween 20 solution (Sigma-Aldrich, St. Louis, MO, USA). Plates were washed five times with 300 µL/well of PBST washing solution [phosphate-buffered saline (PBS), pH 7.4, containing 0.1% Tween 20 (Sigma-Aldrich, St. Louis, MO, USA)], and blocked for two hours at room temperature with 1% BSA-based blocking solution. The blocking solution was removed (without washing), the plates were dried at 37 °C for three hours, stored at 4 °C, and protected from humidity until use. The controls used in this ELISA test included a positive control serum collected from a pig experimentally inoculated with *M. hyosynoviae*, while the negative control was a serum that was collected from animals negative to *M. hyosynoviae*, and other swine mycoplasmas, including *M. hyorhinis*, *M. hyopneumoniae*, and *M. flocculare* [15].

Samples and controls (100 µL/well) were tested, diluted 1:50 in newborn calf serum-based diluent, incubated at 37 °C for one hour, and washed 5 times with PBS-T. Then, 100 µL of HRP-conjugated goat anti-pig IgG (Fc) antibody (Bethyl Laboratories Inc., Montgomery, TX, USA) at a dilution of 1:17,000 was added to each well and incubated at 37 °C for 1 h. After another washing step, the reaction was visualized by adding 100 µL of TMB (Surmodics IVD, Inc., Eden Prairie, MN, USA) solution to each well and stopped by adding 100 µL of stop solution (Surmodics IVD, Inc., Eden Prairie, MN, USA). The absorbance was measured at 450 nm, and results were expressed as sample-to-positive (S/P) ratios.

### 2.9. Multiplex Porcine Cytokine Immunoassay

A 6-plex Luminex assay (ProcartPlex™ Porcine Cytokine panel; Invitrogen, Thermo Scientific, Inc., Pittsburgh, PA, USA) was used to quantify IL-1β, IL-6, IL-8, IL-10, TNF-α, and IFN-γ in serum, following the manufacturer’s instructions. Testing was performed using a Bio-Plex 200 system (Luminex-Diasorin, Austin, TX, USA) operated by Bio-Plex Manager software (Bio-Rad, Hercules, CA, USA). Fluorescence intensity was corrected by subtracting values from the blank wells, and cytokine concentrations were determined using a standard curve generated from the kit’s internal standards [27].

### 2.10. Data Analysis

Statistical analyses were performed to assess differences between groups. The PCR results and the presence of clinical signs and lesions were compared using Fisher’s exact test (*p* < 0.05). Serology, cytokine levels, lesion scores, and leukocyte scores were analyzed using Two-way ANOVA (*p* < 0.05). Statistical analysis and graphical representation were performed in GraphPad Prism version 10.2.2.

## 3. Results

### 3.1. Genomic Comparison Between the Low-Virulence Strain S149 and the High-Virulence Strain 34428

Whole-genome sequencing was performed on the low-virulence strain S149 and the high-virulence strain 34428. The average nucleotide identity (ANI) was calculated using fastANI [28], yielding an ANI of 98.18% between the two strains. Phylogenetic analysis based on the core genome SNPs identified by kSNP3 revealed that they were distantly clustered into separate lineages (Figure 1). Seven genes, *oppF*, *tuf*, *mvsp*, *oppA*, *vamp*, *vapd*, *and MAA1*, were identified as potential virulence-associated factors [9,29,30]. The *oppF* gene was present in the high-virulence strain 34428 but absent in the low-virulence strain S149. In contrast, *tuf*, *oppA*, and *vapD* were detected in both isolates, whereas *mvsP*, *vamP*, *and MAA1* were absent from any isolates included in this study. Although the *tuf* gene was found in both strains, it contained eight nucleotide differences.

### 3.2. Clinical Evaluation

**Figure 2:**
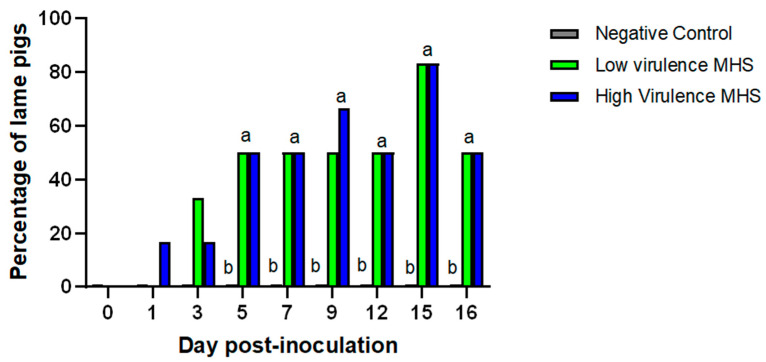
Proportion of lame pigs following *Mycoplasma hyosynoviae* inoculation. The percentage of pigs exhibiting lameness is shown over time post-inoculation. Different letters indicate statistically significant differences between groups (*p* < 0.05).

Lameness, fever, and macroscopic lesions were not observed in control pigs. In contrast, lameness was first detected in one pig inoculated with the high-virulence strain at 1 DPI (Figure 2). From 2 to 16 DPI, lameness was observed in 5 of 6 pigs in both inoculated groups.

Clinically, the severity of lameness did not differ significantly between pigs inoculated with the low- and high-virulence strains, with affected pigs exhibiting mild to moderate lameness. Specifically, three pigs from each inoculated group showed signs of lameness on three or more consecutive days, with scores ranging from 1 to 2. In contrast, the other pigs from both groups were lame for only one or two days, with a score of 1 (Supplementary Table S2). However, the proportion of lame pigs in both inoculated groups was significantly higher than in the negative control group, from 5 to 16 DPI (Figure 2). 

### 3.3. M. hyosynoviae DNA Detection in Tonsil Swabs and Oral Fluids by qPCR

To evaluate *M. hyosynoviae* carriage following inoculation, tonsil swabs and oral fluids were tested by qPCR. All control pigs remained PCR-negative for *M. hyosynoviae* throughout the study.

**Figure 3:**
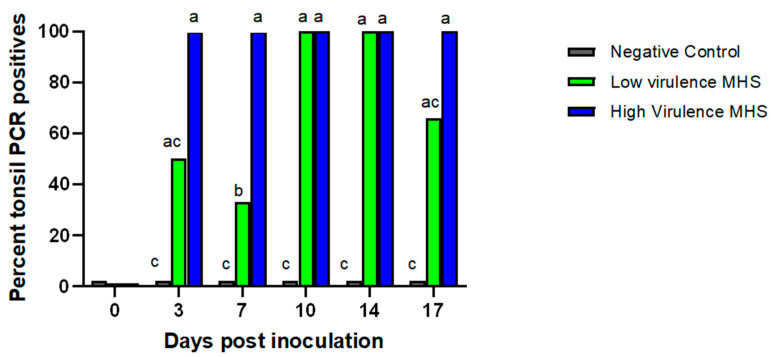
Detection of *Mycoplasma hyosynoviae* in tonsil samples over time. The percentage of PCR-positive tonsil samples collected from pigs is shown by sampling day. Different letters indicate statistically significant differences between groups (*p* < 0.05).

In pigs inoculated with the high-virulence strain, *M. hyosynoviae* DNA was detected in all tonsil swabs from DPI 3 to 17 DPI (Figure 3). At 10 and 14 DPI, all pigs in both inoculated groups tested positive by PCR. The proportion of PCR-positive pigs was significantly higher in the high-virulence group compared to the low-virulence group only at 7 DPI.

**Figure 4:**
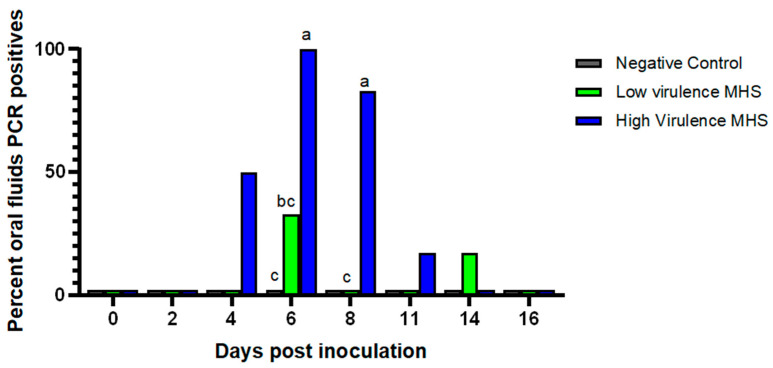
Detection of *Mycoplasma hyosynoviae* in oral fluid samples over time. The percentage of PCR-positive oral fluid samples collected from pigs is shown by sampling day. Different letters indicate statistically significant differences between groups (*p* < 0.05).

Detection in oral fluids was transient, with 3/6, 6/6, 5/6, and 1/6 pigs inoculated with the high-virulence strain testing positive at 4, 6, 8, and 11 DPI, respectively (Figure 4). Pigs inoculated with the low-virulence strain tested positive at 6 and 14 DPI, with only 2/6 and 1/6 positive pigs, respectively. By 16 DPI, all oral fluid samples tested negative.

### 3.4. Post-Mortem Evaluation and M. hyosynoviae DNA Detection in Synovial Fluid by PCR

**Figure 5:**
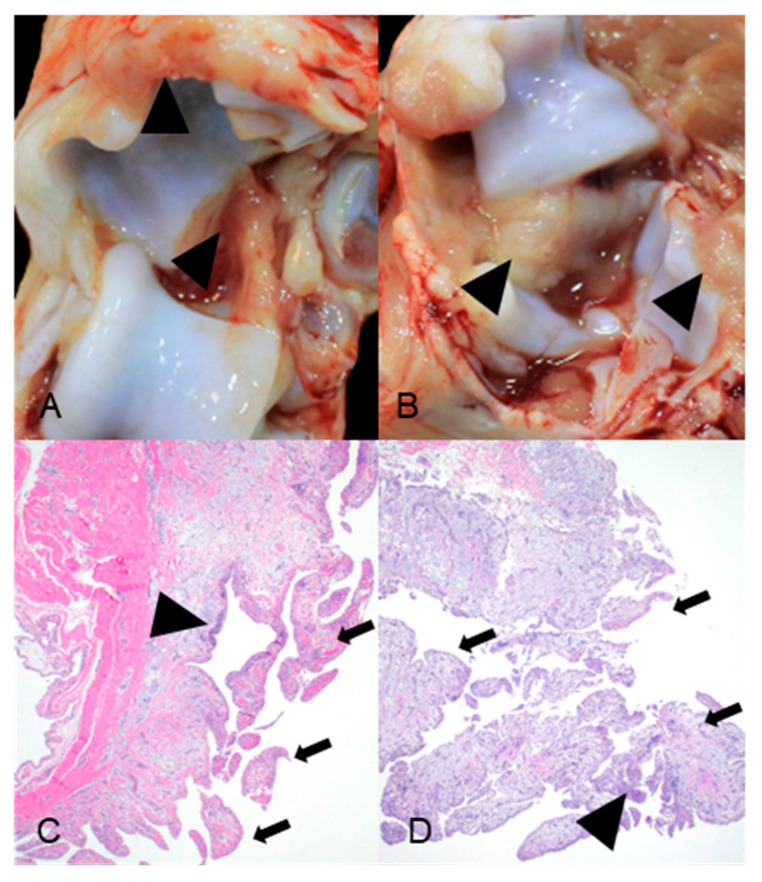
Gross necropsy images (**A**,**B**) and photomicrographs (**C**,**D**) from animal 114 inoculated with *Mycoplasma hyosynoviae* strain 34428 (high virulence) at 18 days post-challenge. (**A**) Synovial hyperplasia and hyperemia (arrowhead) of the tibiotarsal (hock) joint. (**B**) Synovial hyperplasia (arrowhead) of the elbow joint. (**C**) Synovial villous hyperplasia (arrows) and expansion of the subintima by inflammation (arrowhead) of the tibiotarsal (hock) joint. (**D**) Synovial villous hyperplasia (arrows) and expansion of the subintima by inflammation (arrowhead) of the elbow joint.

At necropsy, no joint lesions were observed in control pigs, and synovial fluid from control pigs tested PCR-negative for *M. hyosynoviae*. In contrast, 4 out of 6 pigs inoculated with the high-virulence strain exhibited macroscopic lesions characteristic of arthritis, including synovial hyperemia, hyperplasia, and increased synovial fluid, compared to 1 out of 6 pigs in the low-virulence group. However, this difference was not statistically significant (Table 4, Figure 5).

Microscopic examination revealed that all pigs (6/6) inoculated with the high-virulence strain had one or more joints with lesions consistent with *M. hyosynoviae*-associated arthritis. These lesions included synovial membrane villous hypertrophy, synoviocyte hyperplasia, and mixed inflammatory infiltrates in the synovial subintima. PCR detected *M. hyosynoviae* DNA in all stifle joints, 4 out of 6 elbow joints, and 3 out of 6 hock joints from pigs in the high-virulence group. In contrast, microscopic changes in the control and low-virulence groups were similar, with minimal synovial alterations, including rare perivascular lymphocytes and plasma cells. Additionally, the synovial fluid from all pigs in both the low-virulence and control groups tested was PCR-negative (Table 4).

### 3.5. Leukocyte Counts in the Synovium

The number of leukocytes in the synovium indicates inflammatory or infectious conditions, with elevated counts typically associated with septic arthritis. Pigs inoculated with the high-virulence strain had significantly higher average synovial leukocyte counts than those in the control and low-virulence groups. Although no specific *M. hyosynoviae* joint tropism was evident, leukocyte numbers were highest in the hock joints, followed by the stifle and elbow joints. However, the differences were not statistically significant (Table 5).

### 3.6. M. hyosynoviae Antibody and Cytokine Dynamics in Serum

**Figure 6:**
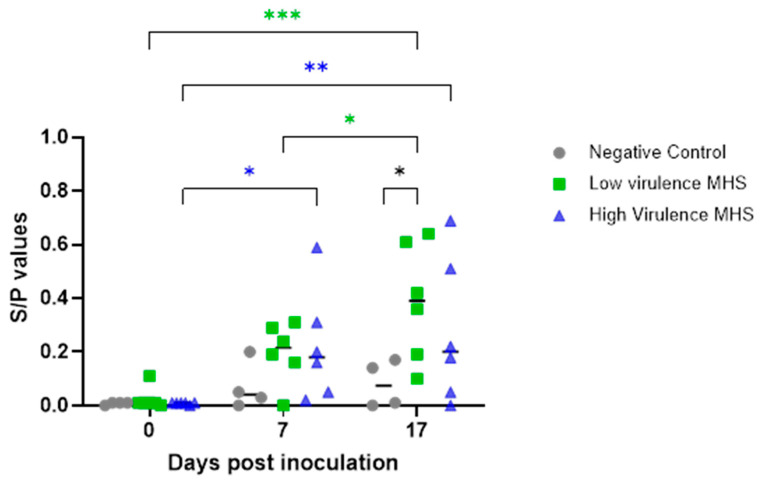
Serum IgG antibody response to *Mycoplasma hyosynoviae* over 17 days post-inoculation. Results are expressed as sample-to-positive (S/P) ratios measured over time. Black lines indicate mean values. Asterisks denote statistically significant differences between groups: black (* *p* < 0.05), green (* *p* < 0.05; *** *p* < 0.001), and blue (* *p* < 0.05; ** *p* < 0.01).

Before inoculation, no serum IgG antibodies against *M. hyosynoviae* were detected. By 7 DPI, a significant increase in serum IgG levels was observed in both inoculated groups, with antibody levels continuing to rise through 17 DPI (Table 6, Figure 6). At 17 DPI, IgG levels in the low-virulence group were significantly higher than in the negative control group.

**Figure 7:**
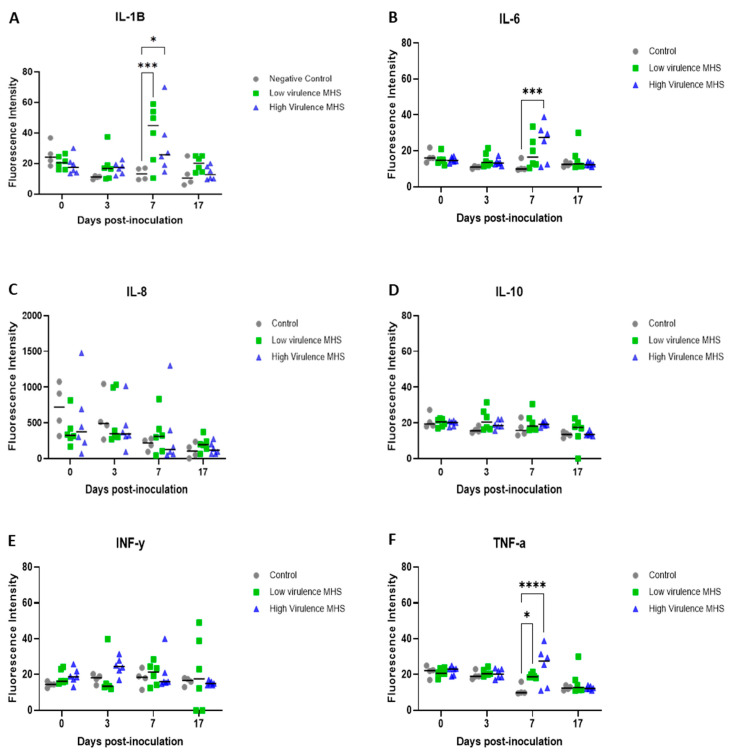
Serum cytokine profiles following *Mycoplasma hyosynoviae* inoculation. Concentrations of IL-1β (**A**), IL-6 (**B**), IL-8 (**C**), IL-10 (**D**), TNF-α (**E**), and IFN-γ (**F**) were quantified over time before and after inoculation. Data are presented as mean ± standard deviation of fluorescence intensity. Asterisks indicate significant differences compared with baseline: * *p* < 0.05, *** *p* < 0.001, **** *p* < 0.0001.

Serum cytokine levels were detected in all pigs before inoculation, and their levels fluctuated throughout the study. The average concentrations of IL-1β, IL-6, and TNF-α peaked at 7 DPI in inoculated pigs relative to controls. More specifically, at 7 DPI, IL-1β and TNF-α levels were significantly elevated in both inoculated groups compared to controls, whereas IL-6 levels were significantly higher in the high-virulence group (Figure 7).

## 4. Discussion

*M. hyosynoviae* is widely distributed in swine populations and is a recognized cause of infectious arthritis and lameness in pigs 12 to 24 weeks [5,6,7,8]. Although tonsil colonization is common, only a subset of colonized pigs develops clinical disease, and the factors influencing systemic spread and arthritis development remain poorly understood [31]. This study investigated clinical presentation, detection dynamics, immune response, and pathology after experimental inoculation with two genetically distinct *M. hyosynoviae* strains, revealing notable strain-specific differences in virulence and immunopathogenesis. Three inoculation routes (IV, IN, and tonsil painting) were utilized for *M. hyosynoviae* inoculation in this study. While IN and tonsil painting are intended to mimic the natural conditions of infection more closely, the IV route was included to reduce variation and enhance arthritis development, following previously published methods [12,15].

### 4.1. M. hyosynoviae Colonization and Detection

*M. hyosynoviae* tonsillar carriage is a critical stage in *M. hyosynoviae* transmission and pathogenesis, as it facilitates bacterial spread within pig populations [5,6,7,8]. Previous studies have indicated that tonsil and oral fluids outperform nasal swabs for detecting *M. hyosynoviae* [12,26]. In this study, tonsil colonization was established by 3 DPI and persisted until the end of the study at 17 DPI, particularly in pigs inoculated with the high-virulence strain. Colonization was detected less consistently with the low-virulence strain. These strain-dependent differences in colonization kinetics may influence transmission dynamics and risk of systemic spread. These findings align with a prior study showing that most sows were naturally colonized at 1 week post-farrowing and remained colonized by 3 weeks post-farrowing [10].

Previous genomic analyses of *M. hyosynoviae* have highlighted substantial variability in genes related to adherence and nutrient acquisition, which may explain differences in colonization efficiency and systemic dissemination between strains [9]. Comparable strain-specific variability in clinical outcomes and virulence has been reported with other mycoplasmas, notably *M. hyorhinis* and *M. hyopneumoniae*. Such variations often relate to differences in surface antigens, metabolic pathways, or immune-modulatory proteins [32]. Given the similarities in genome plasticity across mycoplasma species, it is plausible that analogous genetic mechanisms contribute to the phenotypic variation observed among *M. hyosynoviae* strains.

Oral fluid sampling is a practical, cost-effective surveillance tool for monitoring *M. hyosynoviae* circulation in swine herds [25]. However, in individual pigs in this study, detection in oral fluid samples was transient, with a higher frequency in pigs inoculated with the high-virulence strain. By 16 DPI, all oral fluid samples tested PCR-negative, suggesting that while oral fluids help detect *M. hyosynoviae*, their sensitivity may be lower than that of tonsil swabs when sampled individually. This is consistent with previous findings where pen-based oral fluid sampling from experimentally infected pigs (2 pigs per pen) yielded positive results from 3 to 15 DPI, but subsequent daily sampling until 60 DPI remained PCR-negative [15]. At the herd level, one study linked the proportion of lame pigs in a commercial herd to the frequency of *M. hyosynoviae*-positive pen-based oral fluid samples [33]. However, a direct correlation between the detection of *M. hyosynoviae* in tonsils or oral fluids and *M. hyosynoviae*-associated arthritis has yet to be established, emphasizing the need for cautious interpretation of PCR results, especially in herd-level screening.

### 4.2. Lameness and Joint Pathology

Lameness is a significant animal welfare concern in swine production, with *M. hyosynoviae* being a major infectious cause of arthritis. In this study, mild to moderate lameness was observed intermittently in both inoculated groups. Still, pigs inoculated with the high-virulence strain showed a significantly higher leukocyte count and more PCR-positive joints compared to those inoculated with the low-virulence strain. Notably, histologic lesions consistent with *M. hyosynoviae* and *M. hyosynoviae* DNA were not observed in joints of the group inoculated with the low-virulence strain, suggesting that systemic dissemination and joint infection with this strain may not have occurred or may have been transient and insufficient to cause sustained joint pathology. 

The differences observed between the low- and high-virulence strains’ ability to establish persistent joint infections underscore the importance of strain-specific virulence determinants. Although lameness was observed in pigs receiving the low-virulence strain, the rapid clearance of this strain from joint tissues suggests that certain strains may trigger transient inflammatory responses without persistent infection, possibly due to differences in adherence factors, immune evasion capabilities, or metabolic adaptability. 

Virulence variability among *M. hyosynoviae* strains has been documented in previous studies. In one experimental challenge, two field strains were capable of colonization and arthritis induction, but one strain resulted in more severe disease [12]. A recent core genome MLST analysis of *M. hyosynoviae* isolates from Austrian and German domestic pigs and wild boars revealed substantial genetic diversity [34]. However, further studies incorporating a broader range of *M. hyosynoviae* isolates are necessary to better understand the epidemiological and pathogenic variations in this species.

### 4.3. Virulence-Associated Factors

The genomic analysis performed on the strains used in this study detected the genes *tuf*, *oppA*, and *vapA* in both high- and low-virulence strains, while *oppf* was present only in the highly virulent strain.

The oligopeptide permease proteins (Opp) function primarily as an ATP- and oligopeptide-transport system that brings nutrients into the cell [35]. The genome sequences of seven *M. hyosynoviae* strains revealed a complete Opp operon, comprising the *oppA*, *oppB*, *oppC*, *oppD*, and *oppF* genes [9]. The OppF protein is a component of cytoplasmic ATPases, which provide energy for peptide translocation [9]. Interestingly, the presence *oppF* gene in the highly virulent *M. hyosynoviae* strain could be a factor associated with the higher incidence of lesions and increased detection of the pathogen in the joints of that group. The *oppA* gene was also detected, but it was present in both strains in this study as well as in all strains sequenced in a previous study [9]. The ubiquity of these proteins, encoded by the Opp operon, may reflect the variety of their roles, as they also serve as adhesins, allowing the bacterium to attach to host cells in the tonsils and joints [35].

The *vapD* and *tuf* genes, present in all isolates, could be involved in virulence through different mechanisms. The *tuf* gene encodes Elongation Factor Tu (EF-Tu), which is prevalent among different strains [30]. Studies have shown that *M. hyopneumoniae* and other swine mycoplasmas, such as *M. hyorhinis* and *M. hyosynoviae*, possess an EF-Tu protein that is responsible for binding to one of the host’s own complement regulators, Factor H [29]. Factor H is an inhibitor of complement activation in the alternative complement pathway, binding to host cells to prevent complement-mediated damage and may be one of the mechanisms that mycoplasmas use to evade host immunity [30].

The *vapD* gene is considered a crucial virulence factor in various bacterial species, including *Helicobacter pylori*, *Haemophilus influenzae*, and *Rhodococcus equi*, but its specific role remains unclear [36].

Although *M. hyosynoviae* lacks a cell wall and does not produce classical endotoxins, other unknown virulence factors may contribute to inflammation and joint pathology. Identifying these factors could provide valuable insights into the mechanisms driving arthritis development and aid in designing targeted surveillance, therapeutic, or preventive strategies. 

### 4.4. Immune Response and Cytokine Dynamics

The immune response to *M. hyosynoviae* infection remains poorly understood, particularly the role of humoral immunity. In this study, serum IgG levels increased in inoculated pigs by 7 DPI and continued rising until DPI 17. In addition to intranasal and tonsil painting, the pigs were also inoculated intravenously, which could have aided a systemic infection and consequent humoral response. Seroconversion following natural infection or experimental inoculation has been reported to be variable [5,8,13,15,37]. This variability suggests that while systemic infection stimulates antibody production, tonsillar colonization alone may not be sufficient to induce it. *M. hyosynoviae*-specific IgA antibodies were previously detected in the serum of infected pigs, which were detected later and at lower levels compared to systemic IgG [15]. In that study [15], only a few pigs (three out of ten) developed mild lesions, similar to those observed in our study. Therefore, the correlation between the presence of systemic antibodies and protection from joint colonization or lesion development remains unclear, particularly given that some pigs that were inoculated with the bacteria mounted a humoral response without developing joint pathology. A mixture of cell proteins was used as an antigen in this ELISA test, since there is no recognized specific virulence marker for *M. hyosynoviae*. The development of more specific antigens may be necessary to achieve a better correlation between antibody levels and protection. It has also been demonstrated that *M. hyosynoviae* infection can also induce a strain-specific T-cell response [8], though the functional significance of this remains to be elucidated. 

This study characterized the serum cytokine response following inoculation with *M. hyosynoviae*. Higher levels of IL-1β, IL-6, and TNF-α were detected at 7 DPI in inoculated pigs, with IL-6 peaking in the high-virulence group and IL-1β in the low-virulence group. Similarly, IL-1β and TNF-α were significantly elevated in both inoculated groups compared to controls at 7 DPI. Interestingly, the peak in cytokine concentrations did not align with the most severe clinical signs or lesion burden, suggesting that systemic cytokine elevation may reflect generalized immune activation rather than localized joint inflammation, particularly in response to intravenous inoculation, which resulted in the systemic circulation of the pathogen. Although the pathogen was only detected in the joints of pigs inoculated with the highly virulent strain, cytokine elevation was detected in both groups. Alternatively, differences in cytokine kinetics or cellular sources between strains could explain these patterns.

Proinflammatory cytokines such as IL-1β, IL-6, and TNF-α are key mediators of the innate immune response [38]. *M. hyorhinis* has been shown to induce IL-1α, IL-6, and TNF-α in human monocytes [39] and synovial fluid [40]. These cytokines facilitate early immune activation, as their secretion does not require prior exposure to pathogens [32]. The upregulation of genes mediating these inflammatory cytokines could be correlated with the presence of fibrinosuppurative arthritis in pigs inoculated with *M. Hyorhinis* intraperitoneally [40]. However, the precise role of cytokines in *M. hyosynoviae* pathogenesis remains unclear and warrants further investigation.

In general, the immune response to mycoplasmas is often ineffective in completely eliminating the pathogen, as it can evade the host immune response and persist in colonized tissues [41]. Moreover, local immune responses may also play a role, especially for joint infections, and require further investigation.

## 5. Conclusions

These findings highlight strain-specific differences in the pathophysiology of *M. hyosynoviae*, which may complicate the accurate diagnosis and timely treatment of arthritis in pigs. Colonization can occur rapidly after inoculation, and the carrier state can be detected by qPCR in tonsil and oral fluid samples. However, detection of *M. hyosynoviae* in individual oral fluids appears to be time-dependent. In pigs inoculated with a low-virulence strain, *M. hyosynoviae* was not detected by qPCR in synovial fluid, and microscopic lesions consistent with *M. hyosynoviae* were not observed, despite clinical lameness. This suggests that some strains may not spread systemically as effectively or be cleared from the joints more rapidly, and induce transient joint inflammation. Nevertheless, both strains elicited systemic inflammatory and humoral responses. Future studies should focus on identifying the genetic determinants responsible for phenotypic differences among *M. hyosynoviae* strains, which could enhance our understanding of strain virulence, persistence, and immune interactions. These findings underscore the need for diagnostic approaches and potential vaccine development to consider the substantial strain-to-strain variability in *M. hyosynoviae*.

## Figures and Tables

**Table 1:** Experimental design. *M. hyosynoviae* strains, inoculum concentration, and route of inoculum.

Challenge Strains	Inoculum Concentration	Inoculation Route and Volume (mL)
Negative control	PPLO broth ^a^	Tonsillar painting (2 mL) Intranasal (1 mL) Intravascular (1 mL)
S149 (Low virulence)	10^8^ CFU/mL	Tonsillar painting (2 mL) Intranasal (1 mL) Intravascular (1 mL)
34428 (High virulence)	10^8^ CFU/mL	Tonsillar painting (2 mL) Intranasal (1 mL) Intravascular (1 mL)

^a^ PPLO: Pleuropneumonia-like organism medium (Difco; BD Biosciences, Franklin Lakes, NJ, USA).

**Table 2:** Scoring system used for subjective evaluation of lameness at days −1, 1, 3, 5, 7, 9, 12, 15, and 16 DPI.

Lameness Score	Category	Description
**0**	Normal	Moves freely and uses all four limbs and feet evenly.
**1**	Mild	Shows weight-shifting activities away from the affected limb upon standing, but little or no lameness/adaptive behavior when walking.
**2**	Moderate	Shifts weight away from the affected limb when standing and shows adaptive walking behavior.
**3**	Moderately severe	Reluctant to stand and/or walk; obvious limp and adaptive behaviors when walking.
**4**	Severe	Non-weight bearing on the affected limb when either standing or walking.

**Table 3:** Sample collection schedule for tonsil swabs, serum, and oral fluids.

Sample Type	Days Post-Inoculation
Tonsil swabs	−4, 3, 7, 10, 14, and 17
Serum	−4, 3, 10, and 17
Individual oral fluids	0, 2, 4, 6, 8, 11, 14, and 16

**Table 4:** Pathology (Macroscopic and histopathology) scores and PCR results for joints of all animals.

		Macroscopic *			Histopathology #			PCR		
Groups	Pig ID	Hock	Stifle	Elbow	Hock	Stifle	Elbow	Hock	Stifle	Elbow
Control	135	0	0	0	0	1	0	−	−	−
	129	0	0	0	0	0	0	−	−	−
	116	0	0	0	0	0	0	−	−	−
	113	0	0	0	1	0	1	−	−	−
Low virulent	130	0	0	0	1	0	0	−	−	−
	128	0	0	0	1	0	1	−	−	−
	145	0	0	0	0	0	0	−	−	−
	139	1	0	0	1	1	0	−	−	−
	148	0	0	0	0	0	0	−	−	−
	142	0	0	0	1	1	0	−	−	−
High virulent	118	0	0	0	2	0	0	+	+	+
	132	0	0	0	2	0	1	+	+	+
	137	1	0	0	3	2	0	−	+	+
	150	2	0	2	3	0	2	−	+	+
	114	1	0	2	3	0	3	+	+	−
	125	1	0	0	2	0	0	−	+	−

* Macroscopic score: 0) no visible lesions, 1) mild synovial hyperplasia/hyperemia and/or mild increase in synovial fluid without fibrin, 2) moderate synovial hyperplasia/hyperemia and/or synovial fluid accumulation with or without fibrin, and 3) severe synovial hyperplasia/hyperemia with fibrinous exudate. # Histopathology score 0: Normal to minimal changes; score 1: Mild; score 2: Moderate; score 3: Severe.

**Table 5:** Number of leukocytes (Mean ± SE) in different joint tissues per group.

Groups	Number of leukocytes (Mean ± SE) ^¶^			
	Hock	Stifle	Elbow	Average
Control	17 ± 21.6	14 ± 16.2	17 ± 10.8	16 ± 16.8 (a) *
Low virulent MHS	29 ± 14.2	18 ± 11.7	13 ± 7.1	20 ± 13.2 (a)
High virulent MHS	140/35.6 (A) #	24 ± 35.8 (B)	63 ± 56.1 (AB)	75 ±4.9 (b)

^¶^ The average number of leukocytes observed in the three most severely affected high-power fields (400× magnification) per tissue section. * Lowercase letters: different letters indicate significant differences between groups (rows). # Uppercase letters: different letters indicate significant differences among joints within groups (columns).

**Table 6:** IgG responses (Mean ± SE) in the serum of pigs.

Groups	Day 0	Day 7	Day 17
Negative Control	0.0 ± 0.0	0.0 ± 0.0	0.0 ± 0.0 (a) *
Low virulent MHS	0.0 ± 0.0 (A)	0.2 ± 0.1 (B)	0.4 ± 0.2 (b) (B) #
High Virulent MHS	0.0 ± 0.0 (A)	0.2 ± 0.2 (B)	0.3 ± 0.2 (ab) (B)

* Lowercase letters: different letters indicate significant differences between groups (rows). # Uppercase letters: different letters indicate significant differences within groups (columns).

## Data Availability

The data presented in this study are available upon request from the corresponding author.

## Supplementary Materials

The following supporting information can be downloaded at: https://www.mdpi.com/article/10.3390/pathogens15010066/s1, Table S1: Accession numbers for the *Metamycoplasma (Mycoplasma) hyosynoviae* isolates included in the phylogenetic tree; Table S2: Lameness score assigned to each pig before and after inoculation with *M. hyosynoviae*.
