# Supplementary material for: Clinical Presentation, Detection, and Immunopathogenesis of Mycoplasma hyosynoviae Field Isolates in Experimentally Inoculated Pigs
Source: Pathogens. 2026 Jan 8;15(1):66. doi: 10.3390/pathogens15010066 (PMC12845361; doi:10.3390/pathogens15010066)
Supplement: Supplementary file 1 [file pathogens-15-00066-s001.zip › pathogens-4017455-supplementary.pdf]

Supplementary Table S1: Accession numbers for the Metamycoplasma (Mycoplasma) hyosynoviae isolates included in the phylogenetic tree.

Accession	Sample Name	SPUID	Organism	Tax ID	Isolate	BioProject
SAMN54224688	0449-w1	0449-w1	Metamycoplasma hyosynoviae	29559	2024010449-1	PRJNA1390726
SAMN54224689	0449-w2	0449-w2	Metamycoplasma hyosynoviae	29559	2024010449-2	PRJNA1390726
SAMN54224690	8205-w1	8205-w1	Metamycoplasma hyosynoviae	29559	2024038205-1	PRJNA1390726
SAMN54224691	8205-w2	8205-w2	Metamycoplasma hyosynoviae	29559	2024038205-2	PRJNA1390726
SAMN54224692	8205-w3	8205-w3	Metamycoplasma hyosynoviae	29559	2024038205-3	PRJNA1390726
SAMN54224693	8205-w4	8205-w4	Metamycoplasma hyosynoviae	29559	2024038205-4	PRJNA1390726
SAMN54224694	M-B126-1767	M-B126-1767	Metamycoplasma hyosynoviae	29559	2023109939-1	PRJNA1390726
SAMN54224695	M-B126-1768	M-B126-1768	Metamycoplasma hyosynoviae	29559	2023109939-2	PRJNA1390726
SAMN54224696	M-B28-663	M-B28-663	Metamycoplasma hyosynoviae	29559	2021055790	PRJNA1390726
SAMN54224697	M-B33-755	M-B33-755	Metamycoplasma hyosynoviae	29559	2021055311	PRJNA1390726
SAMN54224698	M-B33-756	M-B33-756	Metamycoplasma hyosynoviae	29559	2021055822	PRJNA1390726
SAMN54224699	M-B45-1121	M-B45-1121	Metamycoplasma hyosynoviae	29559	2022022782	PRJNA1390726
SAMN54224700	M-B66-1252	M-B66-1252	Metamycoplasma hyosynoviae	29559	2022085214	PRJNA1390726
SAMN54224701	M-B82-1377	M-B82-1377	Metamycoplasma hyosynoviae	29559	2023006401	PRJNA1390726
SAMN54224702	M-B93-1450	M-B93-1450	Metamycoplasma hyosynoviae	29559	2023025714	PRJNA1390726

SAMN54224703	M-B94-1467	M-B94-1467	Metamycoplasma hyosynoviae	29559	2023031109-1	PRJNA1390726
SAMN54224704	W7426-1D	W7426-1D	Metamycoplasma hyosynoviae	29559	34428	PRJNA1390726
SAMN54224705	W7426-2D	W7426-2D	Metamycoplasma hyosynoviae	29559	S149	PRJNA1390726
SAMN54198022	M-B181-2095	M-B181-2095	Metamycoplasma hyosynoviae	29559	2025-1	

Supplementary Table S2: Lameness score assigned to each pig before and after inoculation with *M. hyosynoviae*.

Groups	Pig ID	0 DPI	1 DPI	3 DPI	5 DPI	7 DPI	9 DPI	12 DPI	15 DPI	16 DPI
Control	113	0	0	0	0	0	0	0	0	0
	116	0	0	0	0	0	0	0	0	0
	129	0	0	0	0	0	0	0	0	0
	135	0	0	0	0	0	0	0	0	0
Low virulence MHS	130	0	0	1	2	2	1	1	1	1
	128	0	0	0	0	1	0	0	0	0
	145	0	0	0	0	0	0	0	1	1
	139	0	0	0	0	0	0	0	1	0
	148	0	0	1	1	0	1	1	2	0
	142	0	0	0	2	1	1	1	2	1
High virulence MHS	118	0	0	0	1	1	2	1	2	1
	132	0	0	0	0	0	1	0	1	1
	137	0	1	0	1	0	1	2	2	2
	150	0	0	1	2	1	1	2	2	0
	114	0	0	0	0	1	0	0	0	0
	125	0	0	0	0	0	0	0	1	0
